# MUltiparametric Score for Ventilation Discontinuation in Intensive Care Patients: A Protocol for an Observational Study

**DOI:** 10.3390/mps7030045

**Published:** 2024-05-20

**Authors:** Iacopo Cappellini, Andrea Cardoni, Lorenzo Campagnola, Guglielmo Consales

**Affiliations:** 1 Department of Critical Care, Section of Anesthesiology and Critical Care, Azienda USL Toscana Centro, Ospedale Santo Stefano, 59100 Prato, Italy; lorenzo.campagnola@uslcentro.toscana.it (L.C.); guglielmo.consales@uslcentro.toscana.it (G.C.); 2 Department of Anesthesia and Critical Care, Azienda Ospedaliero Universitaria Careggi, 50134 Florence, Italy; cardonia@aou-careggi.toscana.it

**Keywords:** mechanical ventilation, weaning, predictive score, successful extubation

## Abstract

Background: Mechanical ventilation significantly improves patient survival but is associated with complications, increasing healthcare costs and morbidity. Identifying optimal weaning times is paramount to minimize these risks, yet current methods rely heavily on clinical judgment, lacking specificity. Methods: This study introduces a novel multiparametric predictive score, the MUSVIP (MUltiparametric Score for Ventilation discontinuation in Intensive care Patients), aimed at accurately predicting successful extubation. Conducted at Santo Stefano Hospital’s ICU, this single-center, observational, prospective cohort study will span over 12 months, enrolling adult patients undergoing invasive mechanical ventilation. The MUSVIP integrates variables measured before and during a spontaneous breathing trial (SBT) to formulate a predictive score. Results: Preliminary analyses suggest an Area Under the Curve (AUC) of 0.815 for the MUSVIP, indicating high predictive capacity. By systematically applying this score, we anticipate identifying patients likely to succeed in weaning earlier, potentially reducing ICU length of stay and associated healthcare costs. Conclusion: This study’s findings could significantly influence clinical practices, offering a robust, easy-to-use tool for optimizing weaning processes in ICUs.

## 1. Introduction

Mechanical ventilation is a life-saving technique used daily in intensive care units (ICUs) around the world. Nevertheless, this technique is also associated with serious complications and high healthcare costs, often directly linked to its duration [1,2]. At the same time, failure to wean from mechanical ventilation (MV) contributes to unfavorable outcomes, such as longer duration of MV, increased ICU and hospital length of stay and higher mortality. Indeed, cumulative exposure to invasive mechanical ventilation has been associated with further potentially harmful interventions: for example, MV is almost universally related to the administration of large quantities of sedatives [3]. Moreover, prolonged ventilation often results in ventilator-associated complications, long-term functional sequelae and cognitive impairment [4,5,6,7]. For these reasons, weaning from MV represents a crucial step for every patient; it remains essential not to prolong the duration of MV beyond the strictly necessary time [8]. At the same time, identifying the correct timing of extubation or weaning from mechanical ventilation is crucial; in fact, several studies demonstrated that patients requiring reintubation have a higher mortality [9]. Therefore, it is necessary to identify parameters that can predict the ease of weaning from invasive MV. Over the years, several predictive indices have been proposed [10,11,12,13,14,15,16]. The sensitivity and specificity of each depends on the cut-off used. Many of them have good sensitivity, but most have low specificity. When using these indices, it is also important to consider when these values are measured. However, despite these limitations, the systematic use of predictive indices leads to better outcomes compared to clinical judgment alone [17].

## 2. Experimental Design

This is a single-center, observational, prospective, cohort and non-profit study aimed at creating a multiparametric predictive score that could be used as an easy tool to decide the optimal time to discontinue MV in ICUs and to identify patients at high risk of failure. We called this score the MUSVIP (MUltiparametric Score for Ventilation discontinuation in Intensive care Patients). The MUSVIP consists of two assessments performed before and during a spontaneous breathing trial. Given the complexities and the critical importance of timely weaning from mechanical ventilation, we developed the MUSVIP by integrating key ultrasound parameters—diaphragm, heart and lung function—that are interrelated and have been shown to play significant roles in successful ventilation discontinuation. These parameters were chosen based on their demonstrated predictive value in numerous studies assessing readiness for extubation [18]. While other variables could potentially be included, we opted to focus on these core areas due to the stronger body of evidence supporting their relevance and impact on outcomes in weaning processes. Other measurements were not considered for inclusion in the MUSVIP, as existing evidence regarding their predictive utility for weaning success is less conclusive [19].

### 2.1. Setting

This study will be conducted in the 20-bed intensive care unit (ICU) of Santo Stefano Hospital in Prato, which admits over 600 patients annually. Approximately half of these patients require mechanical ventilation for more than 48 h. The enrollment period will last 12 months, with an estimated enrollment rate of 80%, supporting the feasibility of enrolling 219 subjects. The duration of follow-up involves a re-evaluation 28 days after discharge from intensive care.

### 2.2. Study Population

The study population will consist of adult patients admitted to the ICU who are undergoing invasive mechanical ventilation and are candidates for initiating a weaning process.

Every patient who meets the following criteria will be included in the study:

- Informed consent;
- Age > 18 yo;
- Duration of mechanical ventilation > 48 h via endotracheal tube (ETT) or tracheostomy cannula;
- Assisted mechanical ventilation for at least 12 h with FiO_2_ ≤ 0.5, PEEP ≤ 6 cmH_2_O and pressure support ≤ 8 cmH_2_O;
- Respiratory rate ≤ 25;
- PaO_2_/FiO_2_ ratio > 150;
- pH > 7.38;
- Hemodynamic stability;
- Hb > 7 g/dL;
- Normothermia.

The exclusion criteria for this study are as follows:

1. Patients who are unable to provide informed consent and do not have a legal representative available to provide consent.
2. Patients with medical conditions that, according to the clinical judgment of the intensive care team, make the weaning process inappropriate or unsafe.

### 2.3. Bias

In designing this study, several potential sources of bias were considered, and measures were taken to minimize them:

Selection bias: This may occur if the patients included in the study are not representative of the general population of patients undergoing invasive mechanical ventilation. To minimize this bias, we have defined clear and broad inclusion and exclusion criteria. Additionally, all eligible patients in the ICU during the study period will be considered for enrollment.

Information bias: This may occur if the measures of the variables of interest are not accurate or reliable. To minimize this bias, we will use standardized and validated measurement methods for all variables of interest. Furthermore, data will be collected systematically and uniformly for all patients.

Confounding bias: This may occur if the observed association between the variables of interest and the study outcome is influenced by other unmeasured variables. To minimize this bias, we will collect data on a range of potential confounding factors, including demographic variables and comorbidities. These confounding factors will be considered in the statistical analyses.

Follow-up bias (or loss to follow-up): This may occur if patients are lost during follow-up or if there are missing data. To minimize this bias, we will make every effort to ensure complete follow-up of all patients and to minimize missing data.

### 2.4. Sample Size

Based on preliminary data, the AUC for the MUSVIP stands at 0.815. Taking a minimum reference of 0.7 and thus a difference of 0.115 and a calculation performed with R code, the sample size for the present study was calculated by estimating the variance of the AUC using the formula by Hanley and McNeil.

The calculation considered a statistical significance level (alpha) of 0.05 and a statistical power (1-beta) of 0.80. Using the Z approach for estimating sample size, it was determined that 219 subjects are required to validate the score.

### 2.5. Statistical Analysis

The statistical analysis of the collected data will be performed using R software Version 4.4.0; the statistical significance level will be set at 0.05, and all hypotheses will be tested with a two-tailed test. 

The demographic and clinical characteristics of the patients will be summarized using descriptive statistics. Specifically, continuous quantitative variables will be summarized using means and standard deviations, while categorical qualitative variables will be summarized using frequencies and percentages. 

The association between the predictive MUSVIP and the outcome of the extubation process will be analyzed using logistic regression models. The outcome will be considered as a binary dependent variable (success or failure of weaning), while the predictive score will be considered as an independent variable. Odds ratios and 95% confidence intervals will be calculated. Receiver Operating Characteristic (ROC) curves will be used to assess the predictive capacity of the score. The Area Under the ROC curve (AUC) will be calculated to provide an overall measure of the score’s ability to distinguish between patients who are successful in extubation and those who are not. Additionally, ROC curves, through the Youden Index, will be used to identify the optimal cut-off value of the score for predicting the outcome. Correlation matrices will then be calculated to identify which variables within the MUSVIP have a greater impact on predicting the success of ventilation interruption. After a preliminary enrolment, Bootstrap and Monte Carlo simulations will be performed to further define the predictive capability of the MUSVIP. To manage missing data within our study, we will employ multiple imputation techniques, ensuring robustness and integrity in our statistical analysis. This method will allow us to create several plausible sets of data to fill in gaps, thereby preserving the overall data structure and reducing bias introduced by incomplete data.

## 3. Procedure

**Figure 1:**
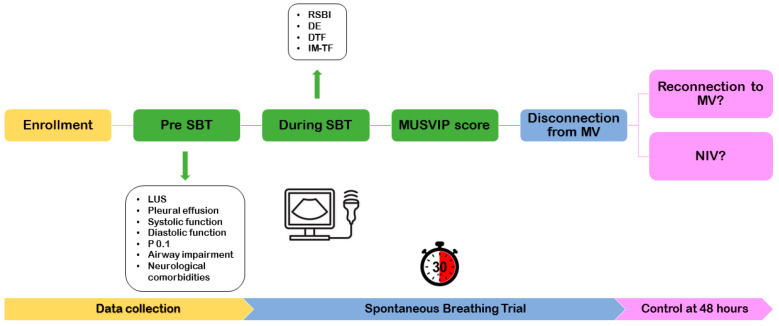
Timeline of the study.

All patients included will undergo a multiparametric evaluation divided into two steps. The first part will be performed before the spontaneous breathing trial (pre-SBT). The second part of the evaluation will be performed during the spontaneous breathing trial. The spontaneous breathing trial will be performed in assisted mechanical ventilation mode by setting PEEP at 0 cmH_2_O and pressure support at 5 cmH_2_O (as suggested by several studies [1]), thus better reflecting the effort required after disconnection from the ventilator and allowing the measurement of tidal volume and respiratory rate during the SBT (Figure 1).

### 3.1. Variables

The demographic variables that will be recorded for the patients included in the study are as follows:

Age: the patient’s age at the time of enrollment in the study.

Sex: the biological sex of the patient.

Ethnicity: the self-reported ethnicity of the patient.

Weight and height: these parameters may be useful for calculating the body mass index (BMI), which can affect respiratory function.

Comorbidities: the presence of other medical conditions (e.g., cardiovascular diseases, diabetes, chronic lung diseases) that could influence the patient’s ability to be weaned from mechanical ventilation.

Duration of mechanical ventilation before the start of the weaning process: this can affect the likelihood of successful weaning.

Reason for intubation and mechanical ventilation: for example, acute respiratory failure, surgery, trauma, etc.

These variables will be collected for each patient at the time of enrollment in the study. The assessment before the SBT involves the measurement of the following parameters:

- LUS (Lung Ultrasound Score);
- Presence/absence of pleural effusion and extent;
- Airway Occlusion Pressure in the first 100 ms (P 0.1);
- Biventricular systolic function (EF for the left ventricle, TAPSE for the right ventricle);
- Left ventricular diastolic function (lateral e′, E/A, E/e′);
- Evaluation of possible impairment of airway protection;
- Neurological comorbidities.

The evaluation during the SBT involves the measurement of the following parameters:

- Rapid Shallow Breathing Index (RSBI);
- Diaphragmatic excursion (DE);
- Diaphragmatic thickening fraction (DTF);
- Intercostal muscle thickening fraction (IM-TF).

The spontaneous breathing trial is set to last 30 min, but will be interrupted if one of the following conditions occurs (resulting in going back to ventilator settings prior to the SBT):

- Respiratory rate > 25/min or <5/min;
- Onset of a new arrhythmia;
- Heart rate > 110 bpm or <45 bpm;
- Systolic Blood Pressure > 160 mmHg or <90 mmHg;
- SpO_2_ < 90%;
- Activation of accessory respiratory muscles;
- Respiratory acidosis;
- Altered mental status.

If the conditions that require an interruption of the spontaneous breathing process do not occur, extubation (in the case of ETT) or permanent disconnection from the ventilator (in the case of a tracheostomy cannula) will be performed. The absence of need for reintubation or reconnection to the ventilator (in the case of a tracheostomy tube) in the 48 h following the spontaneous breathing process will be considered a successful extubation. Ultrasound assessments in our study will be conducted exclusively by one intensivist who is specially trained in lung, muscle and cardiac ultrasonography, ensuring a high degree of consistency and expertise in the measurements.

The outcome variables of this study are as follows:

- Outcome of the extubation process: success or failure.
- Time to successfully complete the weaning process: number of days.
- Length of stay in intensive care: number of days.
- Mortality in intensive care: proportion of patients.
- Need for reintubation within 48 h of extubation: proportion of patients.

For each variable of interest, data will be collected in a standardized manner, and the measurement methods will be the same for all patients. This will ensure the comparability of data across different patients.

### 3.2. Evaluation before SBT

#### 3.2.1. Non-Ultrasound Measurements

The following non-ultrasound measurements will be analyzed:

- Neurological comorbidities (such as ICU-Acquired Weakness, stroke, encephalitis, meningitis, delirium, anxiety syndromes) and the presence of possible factors compromising airway protection (such as neurological deficits, swallowing deficits, ICU-AW) will be extrapolated from patient history.
- The value of the drop in airway pressure 100 milliseconds after the onset of inspiration during an end-expiratory occlusion of the airway (P 0.1) will be derived from the analysis performed by the ventilator.

#### 3.2.2. Ultrasound Measurements

The following ultrasound measurements will be analyzed:

- LUS (Lung Ultrasound Score): The most used score in critical environments for the evaluation of lung parenchyma is certainly the Lung Ultrasound Score (LUS). This scoring system consists of giving a score of pulmonary parenchyma aeration in 12 regions (6 for each hemithorax); the global score is given by the sum of the values of each parenchymal region. In every region, four steps of progressive loss of lung aeration can be identified: score 0 (normal aeration with A lines or no more than two B lines), score 1 (moderate loss of aeration with at least three B lines or confluent B lines or subpleural consolidations involving less than 50% of the visualized pleura), score 2 (severe loss of aeration with confluent B lines or subpleural consolidations involving more than 50% of the pleura visualized) and score 3 (complete loss of aeration with hepatization of the lung parenchyma). As a result, the total LUS value ranges from 0 to 36 [20]. The 12 regions are delimited by anatomical landmarks, as established by consensus in a 2012 conference on point-of-care LUSs [21].
- Over the years, it has been shown that this score reliably evaluates the aeration of the lung parenchyma in critically ill patients [22,23,24,25]. This tool is therefore useful in evaluating lung parenchyma during the weaning process and in identifying patients at high risk of weaning failure. Soummer et al. demonstrated that the LUS predicts weaning failure with accuracy by identifying regional and global lung de-recruitment [26]. In the weaning setting, the LUS has proven accurate in predicting the occurrence of post-extubation respiratory distress: the score is higher in patients experiencing weaning failure and post-extubation distress. On the other hand, the LUS remains significantly below a cut-off value in patients who are successfully weaned. The cut-off identified in the literature to predict an 85% risk of post-extubation failure is >17; the safest value below which the risk of developing a weaning failure is negligible is <13; and a score between 13 and 17 has no predictive value [26];
- Presence/absence of pleural effusion and extent: The accumulation of pleural fluid causes atelectasis due to collapse of the parenchyma adjacent to the effusion, which generates hydrostatic pressure. This can be easily assessed with ultrasound. Large pleural effusions could cause total lobar or pulmonary atelectasis. Atelectasis of lung parenchyma prevents air from reaching the alveoli, thus resulting in less alveolar volume available for respiratory exchanges and the possible creation of a shunt with hypoxemia. Moreover, the lung elastic recoil no longer opposes the elastic return of the rib cage, determining an alteration of the conformation of the latter at the level of the effusion area. The outward movement of the rib cage causes an alteration in the length and tension of the intercostal muscle fibers with lower efficiency of their contraction. The diaphragm is decoupled from the visceral pleural surface, and therefore its contraction presents an attenuated effect on pulmonary insufflation. While the volume of the pleural effusion can be accurately estimated using US [27,28,29,30], it is not clear beyond which volume the drainage is indicated. In our study, the presence and entity of pleural effusion will be evaluated with a convex probe in the lateral and posterolateral thoracic region. The entity of the effusion will be assessed by measuring the maximum perpendicular distance between the parietal pleura and the lung parenchyma.
- Biventricular systolic function (EF for the left ventricle, TAPSE for the right ventricle): To evaluate left ventricular global systolic function, we will use the Ejection Fraction. The EF (%) will be obtained using Simpson’s biplanar method in the apical 4-chamber and apical 2-chamber views (EF = (Left Ventricular End Diastolic Volume − Left Ventricular End Systolic Volume)/Left Ventricular End Diastolic Volume).
- Right ventricular systolic function will be evaluated using Tricuspid Annular Plane Systolic Excursion (TAPSE) in apical 4-chamber view (US beam in M-Mode must be aligned on the lateral portion of the tricuspid annulus obtaining a measurement of its systolic excursion in millimeters).
- In the case of dysfunction of even just one of the two items, the systolic function will be considered abnormal.
- Left ventricular diastolic function (e′, E/A, E/e′): Evaluation of the diastolic function of the left ventricle requires the ability to use Pulsed Wave Doppler (PWD) as well as Tissue Doppler Imaging (TDI). The inflow profile of the mitral valve recorded via the PWD (E wave in the early diastolic phase and A wave in the late diastolic phase) depends on the diastolic function and the filling pressures of the left ventricle, while the TDI of the mitral annulus allows to separate the evaluation of left ventricular relaxation (e′ wave) and left ventricular filling pressure (E/e′ ratio). The use of echocardiography before the SBT could be useful in identifying patients at high risk of weaning failure. In a population of 117 patients, Callie et al. reported that 23 patients who failed the SBT had a lower left ventricle EF and a tendency to higher E/e′ ratios before the SBT [31]. Moschietto et al. reported no difference in a weaning failure group in terms of the LVEF but an increase in the E/e′ ratio [32]. Papanikolaou et al. demonstrated the impact of diastolic function in a group of 50 patients with a preserved LVEF: the worse the pre-SBT diastolic function the higher the percentage of patients with SBT failure [33]. In a meta-analysis by Sanfilippo et al. [34], it was found that diastolic dysfunction and high LV filling pressures were associated with higher percentages of weaning failure, while the role of the LVEF was less clear. In this study, the strongest association with weaning failure was found with high E/e′ values. During the weaning trial, the increased pool of blood returning to the LV may not be easily managed if LV compliance is reduced, creating an increase in post-capillary pulmonary pressures with the onset of pulmonary edema. E wave velocity and the E/A ratio are useful for grading LVDD, but not for its diagnosis. In accordance with the guidelines, in our study, the E and A values will be obtained from the peak velocity of the E-wave and the A-wave (cm/sec) using a PW Doppler in apical 4-chamber view, optimizing the alignment between sampling and blood flow through the color Doppler. The e′ value (lateral) will be obtained in apical projection with 4 cameras using PW-TDI (Pulsed Wave-Tissue Doppler function Imaging) in the lateral basal regions of the LV. From the obtained values, we can measure the E/e′ ratio. To identify diastolic dysfunction, e′ and the E/e′ ratio will be used according to the indications of the 2016 ASA/EACVI guidelines. To distinguish the degrees of diastolic dysfunction (Grade I or Impaired Relaxation, Grade II or Pseudonormalization, Grade III or Restrictive Pattern) the E/A ratio will be used according to indications of the ASA/EACVI guidelines of 2016 [35].

### 3.3. Evaluation during SBT

#### 3.3.1. Non-Ultrasound Measurements

- Rapid Shallow Breathing Index (RSBI): In 1986, Tobin and colleagues, observing breathing patterns during weaning trials, quantified the phenomenon of rapid and shallow breathing with the relationship between the respiratory rate (RR) and tidal volume (TV) [13]. This relationship proved itself superior to all other predictive tests for weaning failure. In fact, currently, one of the most used predictive indices is certainly the Rapid Shallow Breathing Index (RSBI). This index is defined as the relationship between the RR and TV, parameters easily measurable through a spirometric test or simply by using the ventilator. An RSBI ≥ 105 breaths/minute/L indicates that the patient is likely to fail weaning, while a value < 105 is more likely an indicator of possible weaning success. The values of the RR and TV during the SBT will be derived directly from the ventilator monitor in our study.

#### 3.3.2. Ultrasound Measurements

The following ultrasound measurements will be analyzed:

- Diaphragmatic excursion (DE): Ultrasonography allows one to visualize the two hemidiaphragms and their excursions during the respiratory cycle. To evaluate diaphragmatic performance, two different ultrasonographic parameters have been described [36]. The first parameter consists of measuring the diaphragmatic excursion (DE) during inspiration [37]. In the spontaneously breathing patient, diaphragmatic excursion is the result of a given diaphragmatic contraction for a given mechanical load (for example, the compliance of the respiratory system, including abdominal compliance). In patients subjected to positive pressure mechanical ventilation, the diaphragmatic excursion also depends on the extent of the pressure support, such as positive end-expiratory pressure (PEEP). PEEP, in fact, increases end-expiratory lung volume; the increase in this volume lowers the diaphragmatic dome, which may therefore have a reduced excursion [38]. A DE in an SBT < 11 mm increases the probability of trial failure [36]. To evaluate diaphragmatic excursions, a low-frequency convex transducer will be used: it will be positioned in the anterior subcostal region between the midclavicular line and the anterior axillary line. The probe in B-Mode will be positioned in the medial, cranial and dorsal directions in order to evaluate the posterior third of the right and left hemidiaphragms through the hepatic and splenic parenchyma, respectively. The M-Mode sampling line will then be positioned perpendicular to the diaphragm to obtain the maximum cranio-caudal excursion.
- Diaphragmatic thickening fraction (DTF): The second parameter to measure diaphragmatic performance instead describes muscle thickening during inspiration in the area where it is attached to the rib cage [39]. The measurement of diaphragmatic thickening can be used as an index of diaphragmatic efficiency as a pressure generator. The DTF will be measured using B-Mode ultrasonography at the level of the apposition area of the costophrenic sinus (where the diaphragm attaches to the rib cage), that is, anterior to the mid-axillary line near the VII-X ribs. The diaphragm will be identified as a trilaminar structure with two parallel echogenic lines (the diaphragmatic pleura and the peritoneal fascia), which enclose the hypoechoic diaphragmatic muscular structure. The diaphragmatic thickness will be obtained by measuring the distance of the pleural membrane from the peritoneal membrane with B-Mode images of the end of inspiration and the end of expiration. The percentage of the DTF (%) will be calculated with the following formula:
  100 × (end-inspiratory thickness − end-expiratory thickness)/end-expiratory thickness.
  A DTF during an SBT > 30–36% increases the probability of success [36].
- Intercostal muscle thickening fraction (IM-TF): The respiratory muscle pump is made up of 10 different skeletal muscles that are activated depending on the workload imposed [40]. When the diaphragmatic workload increases, these accessory muscles are recruited [41]. With higher levels of diaphragmatic inspiratory support, parasternal muscle activation decreases (in terms of thickening), while in patients with diaphragmatic dysfunction, the parasternal muscle thickening value increases [42]. The IM-TF is a good predictor of weaning outcome in mechanically ventilated critically ill patients. The thickness of intercostal muscles will be obtained using M-Mode ultrasonography. The high-frequency linear probe will be positioned transversely on the sagittal plane at the level of the II-III intercostal space, 3–5 cm laterally to the sternum. Starting in B-Mode, the intercostal muscles will be identified above the pleural line as a trilaminar biconcave structure stretched between two ribs. The muscle thickness will be measured between the hyperechoic layers of the muscle bands. The IM-TF (%) will be calculated with the following formula:
  100 × (end-inspiratory thickness − end-expiratory thickness)/end-expiratory thickness.

### 3.4. MUSVIP: MUltiparametric Score for Ventilation Discontinuation in Intensive Care Patients

The previous parameters will then be integrated into the MUSVIP. A score from 0 to 12 will be assigned to each item (in total, 11 items: 7 items before the SBT and 4 items during the SBT) according to the Table 1 and Table 2 below. The lower the score, the more negative the impact of the item. The total score is a value ranging from 0 to 132. A score will be given to each patient enrolled in the MUSVIP study.

After the SBT, the following categories of patients will be registered:

- Patients who require interruption of their SBT;
- Patients who require reintubation in the 48 h following extubation;
- Patients who require reconnection to the ventilator within 48 h following disconnection from the ventilator (in the case of a tracheostomy tube in place);
- Patients who require non-invasive ventilation in the 48 h following extubation.

## 4. Expected Results

### 4.1. Primary Endpoint

The investigation will primarily focus on the ability of the predictive score to accurately identify patients who will successfully wean off mechanical ventilation. This predictive score is anticipated to serve as a vital tool in streamlining the weaning process by effectively distinguishing between patients likely to succeed and those at risk of extubation failure.

### 4.2. Secondary Endpoints

Alongside the primary focus, the study will examine several critical secondary endpoints to evaluate the comprehensive impact of the predictive score on patient management in the intensive care setting. These include the following:

- Length of stay in intensive care: The study will assess whether the application of the predictive score can contribute to a reduction in the duration of ICU stays. By potentially identifying patients suitable for earlier weaning, the score might help in optimizing ICU resource utilization and enhancing patient throughput.
- Mortality in intensive care: An essential aspect of the study will be to investigate the predictive score’s correlation with patient survival rates within the ICU. This evaluation will help determine if the timely identification of weaning candidates through the score can positively influence patient outcomes.
- Need for reintubation within 48 h of extubation: A crucial measure of ventilation discontinuation success and patient safety is the avoidance of premature extubation, leading to reintubation. The study will explore the predictive score’s effectiveness in reducing the incidence of reintubation within 48 h of post-extubation, signifying a more accurate assessment of patient readiness for weaning.

These outcomes collectively aim to elucidate the predictive score’s effectiveness and utility in enhancing the management of patients undergoing the weaning process from invasive mechanical ventilation. By potentially improving patient selection for weaning, optimizing ICU stays and reducing adverse events like reintubation, the predictive score could significantly impact clinical practices and patient care protocols within intensive care settings.

## Figures and Tables

**Table 1:** Evaluation before SBT.

Variable	Assessment	Partial MUSVIP
LUS	<13	12
	13–17	6
	>17	0
Pleural effusion	Absent or <2 cm	12
	2–4 cm	6
	>4 cm	0
Systolic function	Normal	12
	Abnormal	0
Diastolic function	Normal	12
	Abnormal	0
Neurological comorbidities	Absent	12
	Present	0
Airway protection impairment	Absent	12
	Present	0
P 0.1	≥1 but <5 cmH_2_O	12
	<1 or ≥5 cmH_2_O	0

**Table 2:** Evaluation during SBT.

Variable	Assessment	Partial MUSVIP
RSBI	<105	12
	≥105	0
DE	≥11 mm	12
	<11 mm	0
DTF	≥30%	12
	<30%	0
IM-TF	<10%	12
	≥10%	0

## Data Availability

Not applicable.
